# Hyperglycaemia on admission to hospital and COVID-19

**DOI:** 10.1007/s00125-020-05216-2

**Published:** 2020-07-06

**Authors:** Celestino Sardu, Nunzia D’Onofrio, Maria Luisa Balestrieri, Michelangela Barbieri, Maria Rosaria Rizzo, Vincenzo Messina, Paolo Maggi, Nicola Coppola, Giuseppe Paolisso, Raffaele Marfella

**Affiliations:** 1 Department of Advanced Medical and Surgical Sciences (DAMSS), University of Campania ‘Luigi Vanvitelli’, 80138 Naples, Italy; 2 Department of Precision Medicine, University of Campania ‘Luigi Vanvitelli’, Naples, Italy; 3 Department of Infectious Diseases, Sant’Anna Hospital, Caserta, Italy; 4 Department of Mental and Physical Health and Preventive Medicine, University of Campania ‘Luigi Vanvitelli’, Naples, Italy

**Keywords:** Covid-19, Hyperglycaemia, Inflammatory cytokines, Mechanical ventilation, Non-invasive ventilation

*To the Editor:* We read with great interest the article by Bertrand Cariou and coworkers on people with diabetes hospitalised for coronavirus disease-2019 (COVID-19) [1]. The analysis of the data obtained on a very large population (1317 patients) provided important information, highlighting a role for BMI in disease severity, but did not correctly interpret the data on the role of hyperglycaemia at admission to hospital. In fact, the authors observed an age- and sex-independent association between increased admission plasma glucose and the severity of COVID-19. Nevertheless, they postulate that this observation is rather the consequence of the severity of the infection than a causal primary factor’. This interpretation of the data underestimates the importance of glycaemic control in COVID-19 and may result in caregivers focusing attention on therapeutic management of hyperglycaemic patients. To investigate the importance of glucose management of COVID-19 patients, we analysed recent data on glycaemic control and COVID-19 outcomes. Acute hyperglycaemia occurs in about 50% of patients hospitalised for COVID-19, while the prevalence of diabetes is about 7% [2]. Moreover, increased plasma glucose on admission to hospital was associated with poorer outcomes in patients with mild, moderate and severe COVID-19 [2–4]. Increased concentrations of proinflammatory cytokines, including IL-2 receptors (IL-2Rs), IL-6 and TNF-α, may have a pivotal role in the mechanism(s) underlying this association. Elevated levels of these proteins were common in both COVID-19 and in hyperglycaemia [5–7]. Thus, an over-activation of the innate immune system may be responsible for poorer outcomes in hyperglycaemic patients affected by COVID-19, including those treated with tocilizumab, an inhibitor of IL-6 [7]. Therefore, to date, despite full therapy, including oxygen therapy, hydroxychloroquine and antiviral treatment, hyperglycaemic patients with COVID-19 have a worse prognosis. In this context, we hypothesise that achievement of rapid control of hyperglycaemia in the first 24 h may improve outcomes. To investigate the role of early glycaemic control in the outcomes of patients with COVID-19, we studied 132 consecutive hospitalised hyperglycaemic patients with moderate disease, admitted to Infectious diseases departments (Vanvitelli University, Naples Italy; San Sebastiano Caserta Hospital, Caserta, Italy). Patients were grouped as being with or without severe disease according to post hoc analysis. Hyperglycaemia was defined as plasma glucose level on admission of >7.7 mmol/l. Severity of COVID-19 infection was categorised as follows: mild, patients with fever and no evidence of pneumonia on imaging; moderate, patients with fever, respiratory tract symptoms, pneumonia on imaging without the need for invasive ventilation; critical, occurrence of respiratory failure requiring mechanical ventilation, presence of shock, other organ failure requiring monitoring and treatment in the intensive care unit. Severe disease as a composite endpoint was admission to an intensive care unit, the need for mechanical ventilation or death. Data are presented as mean ± SD, categorical variables were summarised as counts and percentages. According to the post hoc analysis, patients were grouped as being without severe disease (*n* = 72) or with severe disease (*n* = 60). The study protocol was approved by the ethics committee of the University of Campania ‘Luigi Vanvitelli’. Written informed consent was obtained from all patients. There were no significant differences in clinical characteristics between the groups with and without severe disease (age: 64.9 ± 7.1 vs 65.8 ± 6.2 years, *p* = 0.997; BMI: 28.6 ± 1.9 vs 28.3 ± 1.9 kg/m^2^, *p* = 0.877; men: 79.2% vs 84.2%, *p* = 0.263; previous diabetes diagnosis, 19.4% vs 25.0%, *p* = 0.288; insulin treatment on admission in patients with previous diabetes diagnosis without severe disease *n* = 14/50 [28%] vs with severe disease *n* = 15/45 [33.3%], *p* = 0.102; oral glucose-lowering drugs *n* = 43 [86%] vs *n* = 42 [93.3%], *p* = 0.096). When analysing blood glucose levels on admission and after 24 h, we found that a decrease in glucose levels between baseline and 24 h was associated with a lower rate of progression to severe disease and death at 20 days in both nondiabetic and diabetic hyperglycaemic patients (Fig. 1). Thus, to date, early glycaemic control may be a suitable therapeutic option to reduce the poor outcomes in hospitalised hyperglycaemic COVID-19 patients with or without a previous diabetes diagnosis [8].

**Fig. 1:**
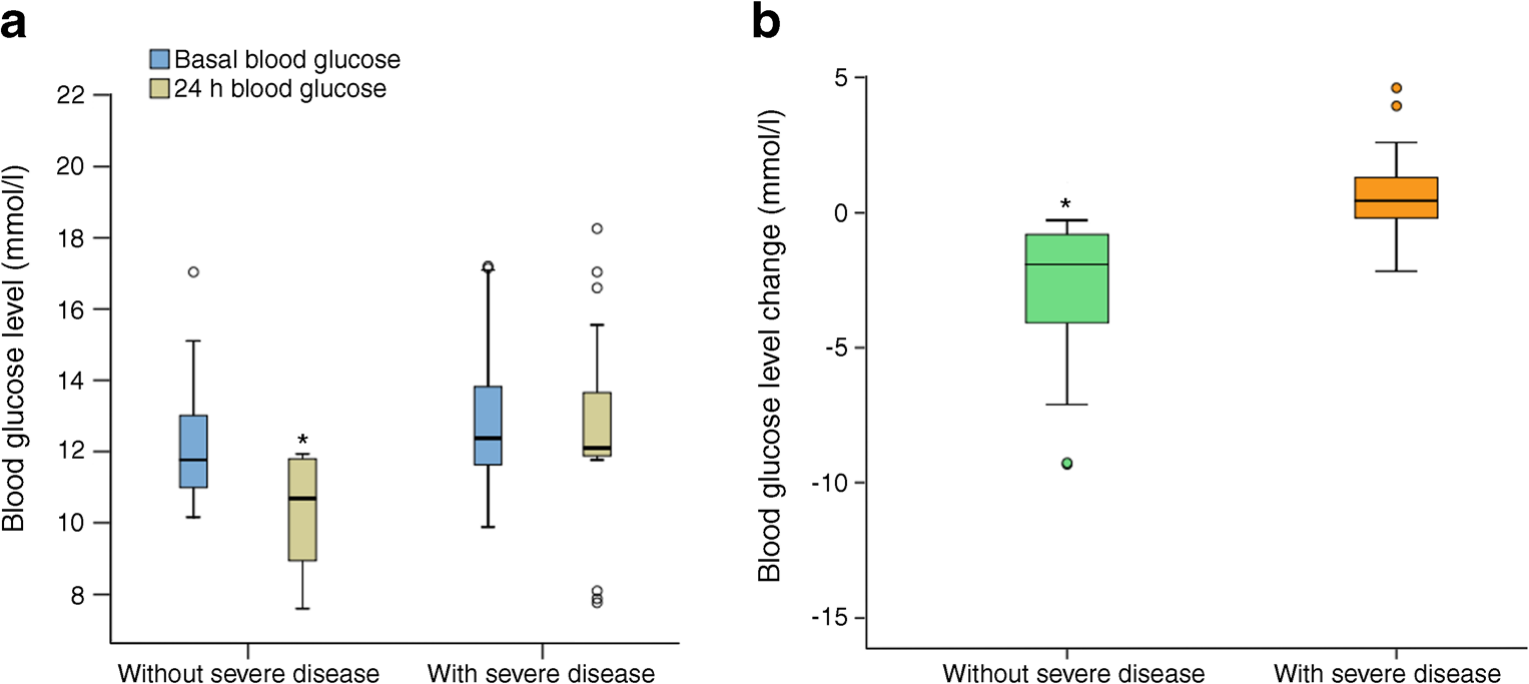
(**a**) Blood glucose levels on admission to hospital and after 24 h for patients with (*n* = 60) and without severe disease (*n* = 72) at 20 days after hospitalisation. (**b**) Blood glucose changes between baseline and 24 h for patients with and without severe disease at 20 days after hospitalisation. Box-and-whisker plots display the median, 25th and 75th percentiles and range. *p < 0.05 by Student’s *t* test; all calculations were performed using SPSS version 23 software (IBM, Armonk, NY, USA)

## Data Availability

The data are available on request from the authors.

## Abbreviation

COVID-19: Coronavirus disease-2019
